# Incineration of Nanoclay Composites Leads to Byproducts with Reduced Cellular Reactivity

**DOI:** 10.1038/s41598-018-28884-y

**Published:** 2018-07-16

**Authors:** Alixandra Wagner, Andrew P. White, Man Chio Tang, Sushant Agarwal, Todd A. Stueckle, Yon Rojanasakul, Rakesh K. Gupta, Cerasela Zoica Dinu

**Affiliations:** 1 Department of Chemical and Biomedical Engineering, West Virginia University, Morgantown, WV 26506 USA; 2 Health Effects Laboratory Division, National Institute for Occupational Safety and Health, Morgantown, WV 26505 USA; 3 Department of Basic Pharmaceutical Sciences, West Virginia University, Morgantown, WV 26506 USA

## Abstract

Addition of nanoclays into a polymer matrix leads to nanocomposites with enhanced properties to be used in plastics for food packaging applications. Because of the plastics’ high stored energy value, such nanocomposites make good candidates for disposal via municipal solid waste plants. However, upon disposal, increased concerns related to nanocomposites’ byproducts potential toxicity arise, especially considering that such byproducts could escape disposal filters to cause inhalation hazards. Herein, we investigated the effects that byproducts of a polymer polylactic acid-based nanocomposite containing a functionalized montmorillonite nanoclay (Cloisite 30B) could pose to human lung epithelial cells, used as a model for inhalation exposure. Analysis showed that the byproducts induced toxic responses, including reductions in cellular viability, changes in cellular morphology, and cytoskeletal alterations, however only at high doses of exposure. The degree of dispersion of nanoclays in the polymer matrix appeared to influence the material characteristics, degradation, and ultimately toxicity. With toxicity of the byproduct occurring at high doses, safety protocols should be considered, along with deleterious effects investigations to thus help aid in safer, yet still effective products and disposal strategies.

## Introduction

Biodegradable polymers such as linear aliphatic thermoplastic polyester^1^ polylactic acid (PLA)^2–4^, made from renewable resources^2,3,5^, have shown good biocompatibility^6–8^ and applicability in food packaging^2^ and medical areas^7,8^. Biodegradable polymers allow for the reduction of environmental risks resulting from high greenhouse gas emissions and fossil fuel energy usage^5^ otherwise encountered at the implementation of conventional petrochemical polymers such as polyethylene (PE), polyethylene terephthalate (PET), polyvinylchloride (PVC), polypropylene (PP), or polystyrene (PS)^5,6,9^. Additionally, since biodegradable polymers require 25–55% less power at their production when compared to the power used to generate petroleum-based polymers^5^, and because of their relatively low production cost resulting from implementation of new processing techniques^6^, it is expected that biodegradable polymers’ usage will increase in the future especially when considering the amount of plastics being needed and/or consumed daily^10,11^. However, such biodegradable polymers, including PLA, are still brittle^6,12,13^ and lack the barrier^4,12^, thermal^4,12^, and impact resistance properties^13^ displayed by the conventional petroleum-based polymers^6^, thus limiting their consumer application.

Recent studies have showed that incorporation of nanoclays, i.e., layered mineral silicates^14,15^ with a platelet thickness of about 1 nm and lengths and widths in the micron range^16,17^, could enhance polymers’ mechanical strength^18–20^, barrier^21,22^, and thermal properties^6,18,23^ when mixed at a low weight percent^16,18^. When such incorporation is attempted, the nanoclays need to be fully exfoliated within the polymer matrix^6^ to allow for increased interactions with the polymer, thus minimizing chain mobility and creating reinforcement effects^18^. For the increased interactions, such nanoclays need to be functionalized with organic modifiers to allow for the required miscibility within the polymer^24,25^, as well as a better incorporation/exfoliation^19^. One example of a nanoclay isolated from the clay fraction of soil^14,15^ is montmorillonite (MMT) which can be easily modified with methyl, tallow, bis-2-hydroxyethyl, quaternary ammonium (to form Cloisite 30B (CC)) for facile incorporation within PLA^1,19,26,27^. The good miscibility observed upon such nanoclay incorporation is presumably due to interactions of the C=O moieties present in PLA with its modified hydroxyl groups^1^. Due to the resulting increased barrier properties^20,28,29^, UV dispersion^21,30^, transparency^31^, mechanical strength^28,32^, and a longer shelf life^17^, polymer-based nanoclay nanocomposites have shown increased implementation in food packaging with the ability to withstand physical stresses associated with transportation and handling^33^. Further, PLA-CC nanocomposites were shown to provide a “green” packaging material that has a lower environmental impact and increased sustainability relative to conventional polymers^1,12,17^. Upon the end of their use, such nanocomposites are known to either be disposed in the landfills, incinerated, or recycled^34,35^. However, due to plastics relatively high stored energy value^11^, the PLA-based nanocomposites make good candidates for disposal via municipal solid waste (MSW) plants, with the waste being combusted to allow for the recovery of energy and reduction of volume of waste of up to 90%^11^.

Considering the large implementation that is envisioned for such nanocomposites, recent research is focused on determining whether they have toxicological profiles. The need to identify possible deleterious pathways is driven by the minimal studies on their toxicity in both manufacturing and disposal areas, with the available toxicity studies only considering the migration extracts from such nanocomposites^36,37^, and other numerous results showing that nanoclays by themselves can induce toxic effects upon exposure to lung cells^38–41^ in such areas^42–44^. Specifically, Maisanaba *et al*. examined the toxicity of migration extracts from a PLA-Clay 1 (a nanoclay modified with hexadecyltrimethyl-ammonium bromide (HDTA)) and PLA-Clay 2 (a nanoclay modified with HDTA and acetylcholine chloride) nanocomposite on Caco-2 and HepG2 cells and found no significant toxic effects^36^. Similarly, Maisanaba *et al*. examined the toxicity of a PLA-Clay 1 migration extract on Wistar rats and found no significant toxic effects^37^. However, Zia *et al*. examined the toxicity of nanocomposite films via investigation of cell attachment and spreading of L-929 cells on a chitin based polyurethane-bentonite nanocomposite and found that nanocomposites with increasing amounts of bentonite had adverse effects on the samples’ biocompatibility with less adhesion and dissimilar morphology of the cells relative to control cells^45^. Complementary, we and others showed that nanoclays by themselves decrease cellular proliferation^38,40^, cause mitochondrial^46^ and membrane damage^46,47^, induce reactive oxygen species (ROS) generation^46^, and genotoxic effects, such as micronuclei induction^48,49^ and changes in mRNA expression^48^.

Considering that ultrafine and fine-sized particles could result from disposal of nanocomposites via MSW plants to potentially escape exhaust filters^42^, and that the high temperatures encountered in the MSW disposal^42^ could cause property changes of the incinerated material^50,51^ to create a byproduct with its own toxicological profile^40,52^, we aimed to determine the toxicity of incinerated PLA-CC nanocomposites through the use of a model *in vitro* cell line, human bronchial epithelial (BEAS-2B) cells^53^. The toxicity of such thermally degraded nanocomposites (i.e., herein called byproducts) is expected to allow for correlation studies between the consumption/usage and disposal stages during the nanocomposite’s life cycle, while also ensuring the individual toxicological impacts and material characteristics of the components themselves, i.e. PLA and nanoclay, as well as their associated byproducts, are explored. Such a study could potentially lead to mitigation strategies for worker protection and controlled land field disposal of byproducts to minimize bio-interactions.

## Results and Discussion

Considering that nanocomposites (or nanoclay melt-mixed within polymers) have seen increased implementation in food packaging^54,55^, with such products being disposed by incineration because of their energetic costs reduction and cost recovery^11,56^, we aimed to design a platform for meaningful assessment of possible toxicity profiles. The need for toxicity studies is driven by the recent reports that show that nanoparticles resulted from incineration have the potential to escape filters in disposal areas^42,57^ and induce toxic effects on the lung^40,41^ of the workers present in such environments. However, no reports are existing that assess human exposures in such areas.

To demonstrate the feasibility of the designed platform, we used a model polylactic acid (PLA)-based nanocomposite since PLA has seen a high consumer implementation in the food packaging industry^58,59^ due to its known “green polymer” characteristics and granted approval by the Food and Drug Administration^32,60^. In the first part of the assessment strategy, we evaluated materials’ and byproduct resulting from incineration characteristics, while in the second we assessed any induced deleterious effects of such byproducts on model human lung cells and correlated the observed toxicological mechanistic profiles with the starting material or resulting byproducts physico-chemical characteristics.

We first created the PLA-based nanocomposite (PLACC) by melt mixing Cloisite 30B (CC) into PLA^61–64^. Films formed from solely PLA served as controls. Consideration was given to CC as a model nanoclay because of its good miscibility in PLA^4^, large consumer implementation^65,66^, and the available reports on its toxicity on systems such as liver^67^, colon^68^, and lung^40^, where it has shown both reductions in proliferation and viability^40,67,68^, as well as cellular membrane damages^67^, changes in cellular morphology^40^, and increased reactive oxygen species (ROS) generation^69^.

We then aimed to mimic the route of disposal by incineration of such nanocomposites using conditions encountered in MSW plants^42^. Specifically for this, we thermally degraded both PLACC and the PLA control films under temperatures ranging from 25 to 950 °C and then evaluated the resulting moisture, volatile, and ash contents. As expected, no ash was obtained upon PLA films incineration, indicating complete degradation of the polymer (Table 1). However, PLACC had around 4% of its weight remaining as ash, likely due to CC, which was added at 5 wt. %. Additionally, PLACC had a significantly lower amount of volatile content relative to PLA, again, presumably due to the presence of such nanoclay. Our results are supported by Koh *et al*. who also showed byproduct formation after degradation at up to 700 °C of PLA containing either Cloisite 15A or Cloisite 20A respectively^70^. However, the ash content identified in our study was larger than the previous one, most likely because of a more prominent char resistance of the CC relative to the other nanoclays^70^ as dictated by their different thermal stability resulted from their respective organic modifier composition (i.e. amount of volatile compounds present) and the wt. % in which the organic modifier was added to them^41^. Specifically the organic modifiers for Cloisite 15A or 20A respectively are made up of 2 tallow groups when compared to CC which is made up of only one tallow group (Scheme S1)^70^.

**Table 1:** The amount of moisture, volatile, and ash present in PLA and PLACC as determined by TGA. The symbol * indicates a significant difference between PLA and PLACC (n = 4).

	Moisture	Volatile	Ash
PLA	0.49 +/− 0.18	99.70 +/− 0.27	0 +/− 0
PLACC	0.60 +/− 0.17	95.37 +/− 0.43*	3.92 + /− 0.10*

**Figure 1:**
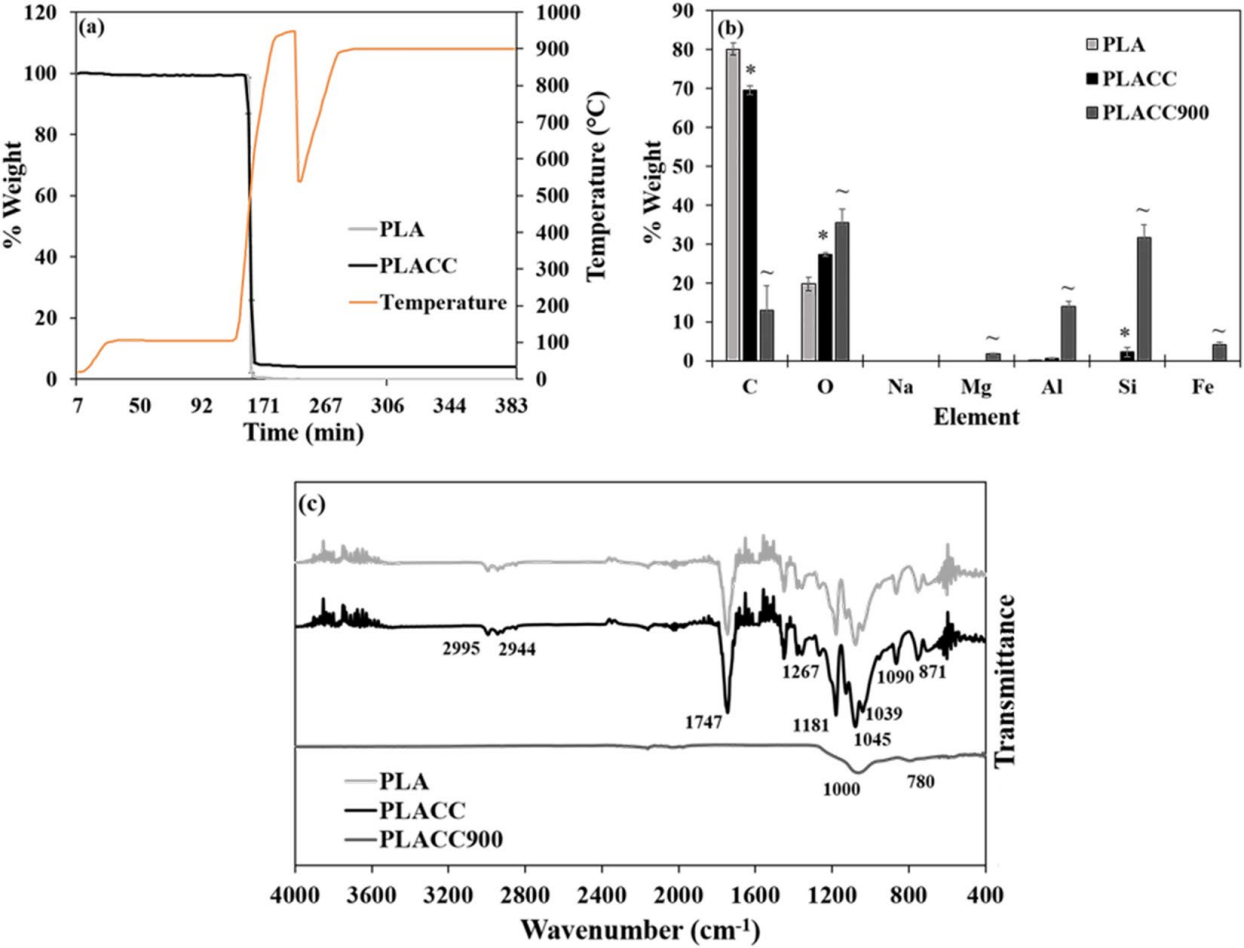
(**a**) Thermal degradation profile of PLA and PLACC as determined by TGA (n = 2). Chemical characteristics analysis. **(b**) Elemental composition of PLA, PLACC, and PLACC900 as determined by EDX (n = 5; taken from 5 different areas on the sample). The symbol * and ~ indicate significant differences between PLA and PLACC and between PLACC and its incinerated byproduct, PLACC900, respectively. (**c**) FTIR spectra for PLA, PLACC, and PLACC900 (n = 2).

Based on our analysis, both PLA and PLACC lost the majority of their weight in the range of 300–600 °C, with PLACC showing a slightly slower degradation rate relative to control PLA (Fig. 1a). Overall, the differences in degradation rate and onset degradation temperature were fairly minimal between PLA and PLACC, showing that the addition of CC did not appear to significantly influence PLA’s thermal stability. It is known that thermal stability of nanocomposites is generally dependent on the degree of dispersion and wt. % of the nanoclays, with a well exfoliated nanocomposite displaying increased stability^71,72^. This is presumably due to the thermal stability of inorganic materials^70^, their interactions with the polymer substrate that allow for the formation of char by hindering the release of volatile products^70,72,73^, or/and to the nanoclays themselves which could potentially be creating a protective barrier when on the surface of the nanocomposite^71^.

The nanocomposites, control films, and their byproducts resulting from incineration were subsequently investigated for their chemical (elemental and molecular compositions) and physical (morphology, mechanical and optical properties, crystallinity and degree of exfoliation of CC in PLA, and hydrodynamic diffusion versus projected area of byproducts, respectively) characteristics.

For chemical characteristics specifically, the elemental composition of PLA, PLACC, and PLACC900 was determined by energy dispersive X-ray (EDX) spectroscopy. Analysis confirmed the presence of carbon and oxygen as the majority of the elements for PLA (Fig. 1b), as well as a significant decrease in carbon content and increase in oxygen and silicon contents respectively in the PLACC nanocomposite presumably resulting from the incorporation of the CC^14^. Upon thermal degradation, the amount of carbon was significantly decreased, confirming the loss of PLA^74^. Additionally, PLACC900 had a significantly higher amount of oxygen, magnesium, aluminum, silicon, and iron, all relative to PLACC with such elements being associated with the presence of the nanoclay itself^14,75^, thus signifying that the ash content was made up mostly of the nanoclay byproduct.

Molecular composition of the nanocomposites, PLA control films, and PLACC900 was determined by Fourier Transform Infrared Spectroscopy (FTIR). PLA and PLACC both displayed similar spectra (Fig. 1c), as previously reported for PLA itself^21,76–78^. Specifically, both PLA and PLACC displayed peaks at 1267, 1181, 1090, and 1045 cm^−1^, indicative of -C-O- stretching^76,77^ and at 1454, 1384, and 1362 cm^−1^, indicative of symmetric and asymmetric deformational vibrations of C-H present in the CH_3_ groups of the PLA respectively^21,76–78^. Additionally, the peaks present at 2995 and 2944 cm^−1^ and 1747 cm^−1^ were indicative of -CH-^76–78^ and C = O stretching^21,77,78^, respectively. Finally, the peak at 871 cm^−1^ was presumably due to -C-C bond formation^76,77^. Peaks specific for CC did not show up in PLACC likely due to the low concentration at which this nanoclay was added when the nanocomposite was formed. Similar results were obtained by Moo-Espinosa *et al*., when CC was exfoliated into segmented polyurethanes at concentrations of 2, 6, or 10 wt. %, respectively^79^.

All of the peaks associated with PLA were no longer present for the byproduct, PLACC900, confirming the degradation of the polymer upon nanocomposite’s incineration. The only 2 peaks remaining for PLACC900 were associated with Si-O-Si stretching vibration of silicate as indicated by the peak around 1000 cm^−1^ ^46,80^, and Si-O indicated by the peak observed around 780 cm^−1^ ^81^. Along with the loss of polymer, the nanoclay itself also lost its organic modifier as confirmed by the absence of peaks at 2920, 2850, and 720 cm^−1^ ^46,80–82^. Further, the loss of the alumino-silicate lattice normally displayed by MMT was confirmed by the loss of peaks associated with Al-OH-Al deformation (900 cm^−1^)^46,80^ and OH respectively which was previously linked to Al^3−^ and Mg^2−^ (840 cm^−1^) (Supplementary Fig. S1)^81^.

For physical characteristics we considered that thermal degradation led to an ash byproduct, as such we only investigated the crystallinity of PLA and PLACC, as well as the degree of exfoliation of CC within the polymer, by using X-ray diffraction (XRD) in the 2θ ranges of 5–80° and 1–10°, respectively. In the context of our goal to design a platform for meaningful assessment of toxicity, crystallinity was to be evaluated since it has been previously shown to influence toxicity^83–85^, with crystalline materials being known to produce oxidant species with pronounced deleterious cellular effects^83,85^. Also, exfoliation of nanoparticles has previously shown to influence toxicity, with studies showing that toxicity generally decreased when nanoparticles were properly exfoliated versus when they were in agglomerate forms^86,87^. Further, both crystallinity and exfoliation have been shown to influence degradation of materials^64,87,88^, which in itself could potentially cause for a differential change in deleterious effects^89^.

**Figure 2:**
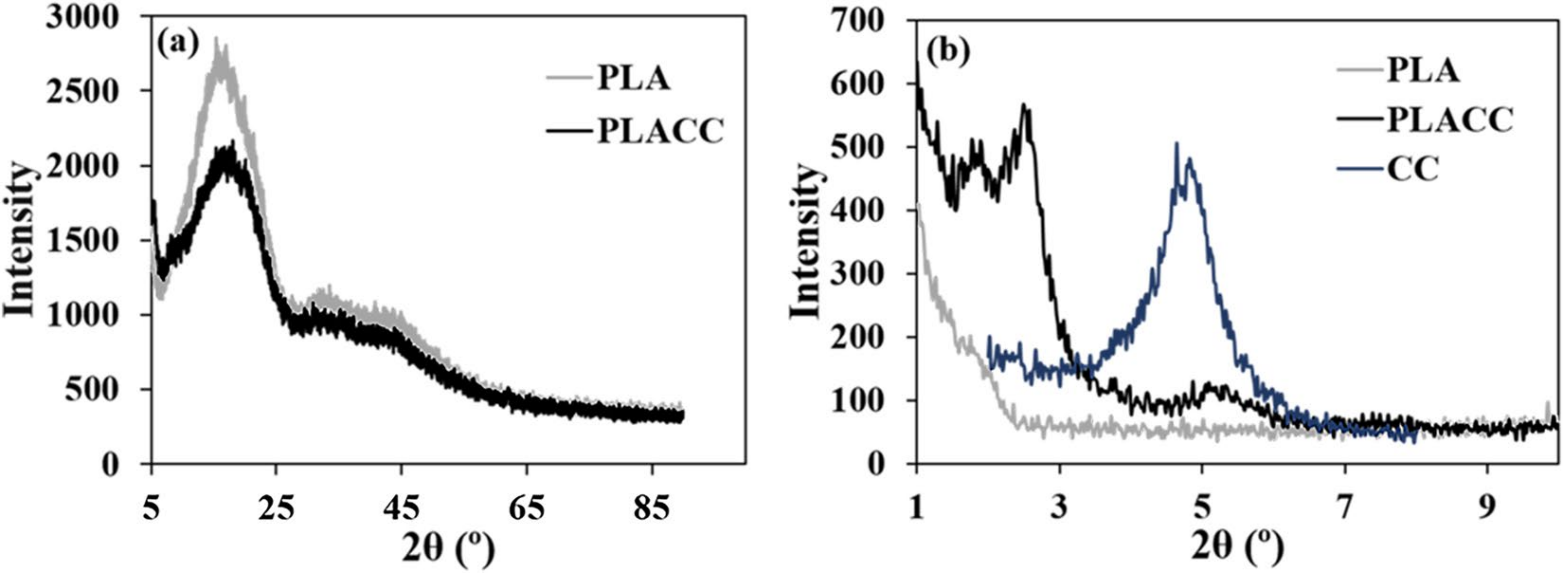
Physical characteristics analysis. (**a**) Crystallinity of PLA and PLACC as determined via XRD. (**b**) Exfoliation of CC in PLACC as determined via XRD.

Our analysis showed that in the 2θ range of 5–80°, both PLA and PLACC displayed broad peaks around 15.4 and 18.1°, respectively (Fig. 2a), which are characteristic of neat PLA^90^ thus confirming the amorphous structure and low crystallinity of the samples^79,91^ likely induced by the high cooling rates used during the molding process of the polymer^92^.

No peaks were observed for PLA in the 1 to 10° 2θ range which was in contrast with CC and PLACC which both displayed peaks within that range (Fig. 2b). Specifically CC, displayed a peak at around 4.8°, presumably indicating a basal spacing of 1.85 nm^93^. This peak was also present for PLACC, however at a lower intensity, presumably demonstrating that a small amount of the nanoclays were likely agglomerated within the polymer matrix^12^. Additionally, PLACC displayed peaks at smaller angles, i.e., around 1.8° (basal spacing of 4.92 nm) and 2.5° (basal spacing of 3.54 nm) respectively, presumably due to the penetration of the polymer chains between the nanoclay platelets and that resulted in increased basal spacing to confirm intercalation or exfoliation of the nanoclay within the PLA^12,94–96^.

**Figure 3:**
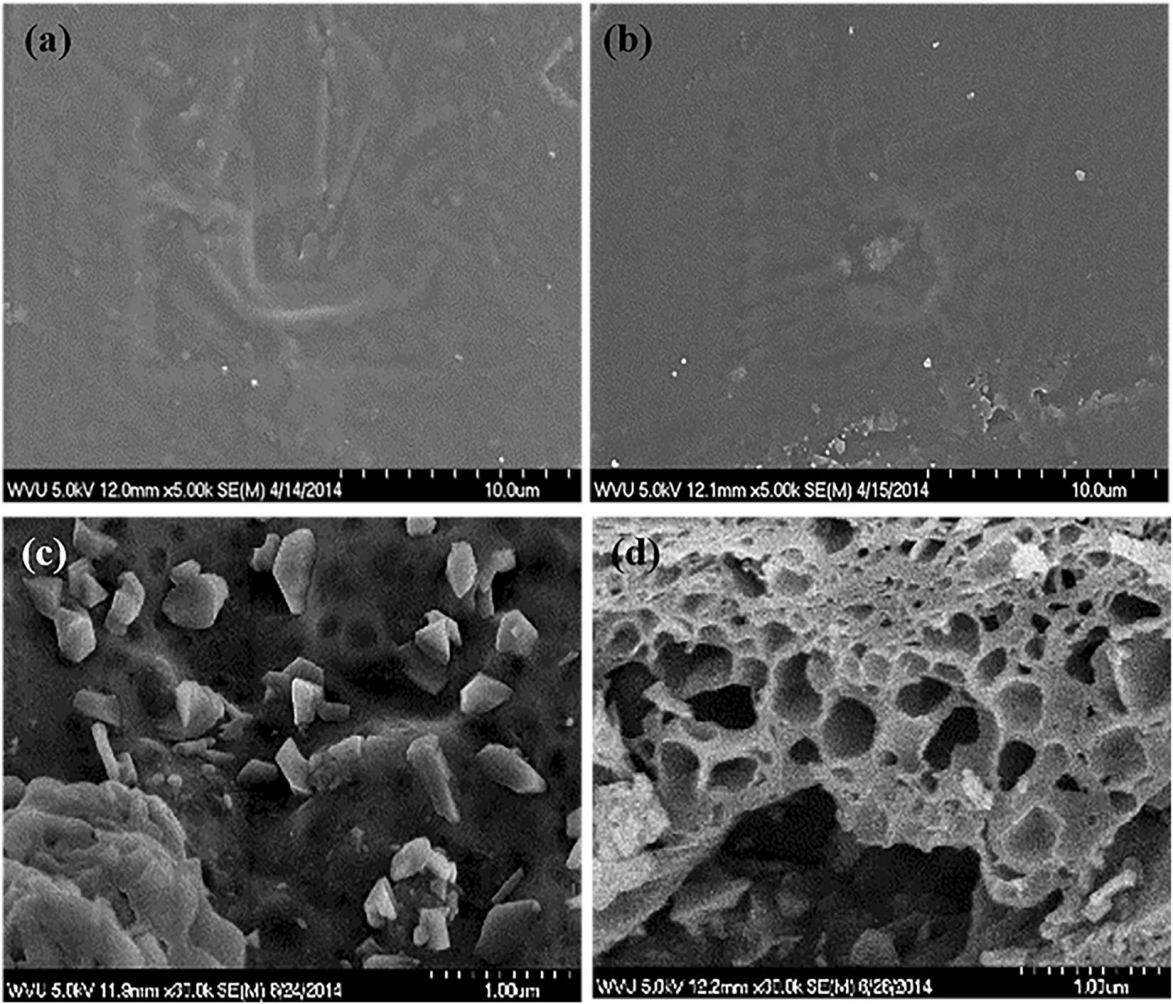
Surface morphology of (**a**) PLA, (**b**) PLACC, and (**c**), (**d**) the two morphologies displayed by PLACC900 as determined by SEM.

The CC did not seem to be completely surface exfoliated within PLA^96^, as confirmed by surface morphology analysis performed by scanning electron microscopy (SEM). Specifically, results showed that PLACC displayed a slightly rougher morphology relative to PLA used as control (Fig. 3a,b). Complementary, upon nanocomposites degradation, PLACC900 displayed generally two types of morphologies, namely one with a fragmented surface with platelets jutting out, and a second one with a porous conformation (Fig. 3c,d). Such different morphologies may be due to differential distribution and degrading of the nanoclay within the polymer matrix, interactions of the nanoclay with the polymer, and/or the different exfoliation noted. Degree of dispersion can be controlled in the future by manufacturing parameters, such as, temperature^97^, time^22^, and feed rate^98^. Previous results by Stueckle *et al*., showed a porous morphology if only CC was degraded, with the degraded CC’s (CC900) morphological changes being attributed to the interactions of the organic modifier with Si-O and Al-O bonds in the pristine clay and an increase in basal spacing of the nanoclay^52^. The porous morphology of the degraded nanocomposite, PLACC900, could also be attributed to the polymer increasing the basal spacing between CC, with the fragmented morphology potentially due to agglomerated CC. Indeed, our control experiments of thermally degraded CC (CC900) showed that the main difference between the thermally degraded nanocomposite (PLACC900) and CC900 respectively, appeared to be related to the physical properties and not changes in the elemental or molecular properties of the two samples (Supplementary Fig. S1).

Additional physical characterizations of transparency and UV dispersion of PLA and PLACC provided further insights into the exfoliation of CC into PLA. Both means of characterizations have previously been shown to be contributing to understanding physical properties and implementation as they allow for a consumer “to see the product” and for the blocking of light/UV transmission to increase product’s shelf life^99^ by reduction in the UV driven lipid oxidation and discoloration^100^. Analysis showed that PLA and PLACC displayed similar absorbance spectra with peaks around 245 and 270 nm, respectively (Supplementary Fig. S2). PLACC also had a significantly higher transparency than PLA (Supplementary Table S1) which could indicate a better orientation upon addition of CC in the nanocomposite volume^12,101^ since previous analysis showed that cast control films typically have a low degree of crystallinity and transparent appearance due to the rapid cooling^74^. Complementary, both PLA and PLACC generally displayed good UV dispersion properties with around 4 and 3% transmittance, respectively. The slight decrease in PLACC’s UV dispersion relative to PLA was most likely due to the presence of the nanoclays which are known to enhance the scattering of the UV light^102^. Additionally, upon incorporation of CC, PLACC displayed a color change (to a brown color, Supplementary Fig. S2), further known to be preferable for preventing UV transmission in food packaging^103^.

Mechanical properties analysis of the nanocomposite showed that PLACC had a significantly higher Young’s Modulus relative to PLA films, thus indicating that CC interacted with the polymer within the volume of the nanocomposite (Supplementary Table S2)^71^. However, both the elongation and the tensile strength were lower for the nanocomposites when compared to control films of PLA, presumably due to an uneven dispersion of CC with reduction in chain mobility caused by dispersed nanoclay^104^ and the reduction in strength caused by agglomerated or poorly dispersed areas containing nanoclay^105^. Further, such agglomerated nanoclays caused for poor interfacial bonding between the nanoclays and polymers, leading to the formation of microcracks in such areas^104,106^, as well as lower plasticity^107^, thus confirming uneven distribution of CC, with likely both agglomerated and exfoliated particles, as previously shown for the PLACC nanocomposite.

Lastly, the hydrodynamic diffusion versus projected area of byproducts was evaluated via dynamic light scattering (DLS) since previous analysis have showed that the size of a particle could influence its toxicity^108^ and internalization profiles^108^. Specifically, spherical particles less than 10 µm can be inhaled^109,110^ with particles smaller than 2.5 µm potentially reaching the alveoli^109^. Further, particles of up to 25 µm in diameter were shown to be deposited in ciliated airways if they had a platelet like morphology and a thickness of less than 0.1 µm^111^. In our investigations, a cellular relevant media and a control buffer solution (PBS) were used. Analysis showed that PLACC900 displayed size distributions in the micrometer range in both media and control buffer, PBS (Supplementary Fig. S3). Specifically, 90% of the particles were under 13 µm, with 50% of such particles being under 5 µm, respectively (Table 2). The lack of difference in size distribution between the 2 chosen solutions was presumably due to the recorded loss of the organic modifier from CC and the majority of the polymer matrix^41^. Specifically, the presence of the organic modifier has shown to cause for size differences between organically modified clays^41^ due to interactions of the modifier with proteins in the media, with differences dependent on the make-up of the organic modifier and its relative hydrophilicity. However, once the organic modifier was lost, these size differences between the nanoclays no longer existed^41^.

**Table 2:** Average particle size distributions (µm) of PLACC900 in cellular media and control buffer, PBS (n = 3).

	PBS	Media
<10%	3.00 +/− 0.01	3.12 +/− 0.08
<50%	4.57 +/− 0.01	4.84 +/− 0.29
<90%	12.67 +/− 0.03	12.14 +/− 1.14

Thermally degraded PLACC byproduct (i.e., PLACC900) was exposed to model human bronchial epithelial (BEAS-2B) cells. BEAS-2B cells were chosen for inhalation toxicity assessment due to their ease of handling and incorporation in numerous studies evaluating toxicity of nanoparticles via the route of inhalation^112–115^.

**Figure 4:**
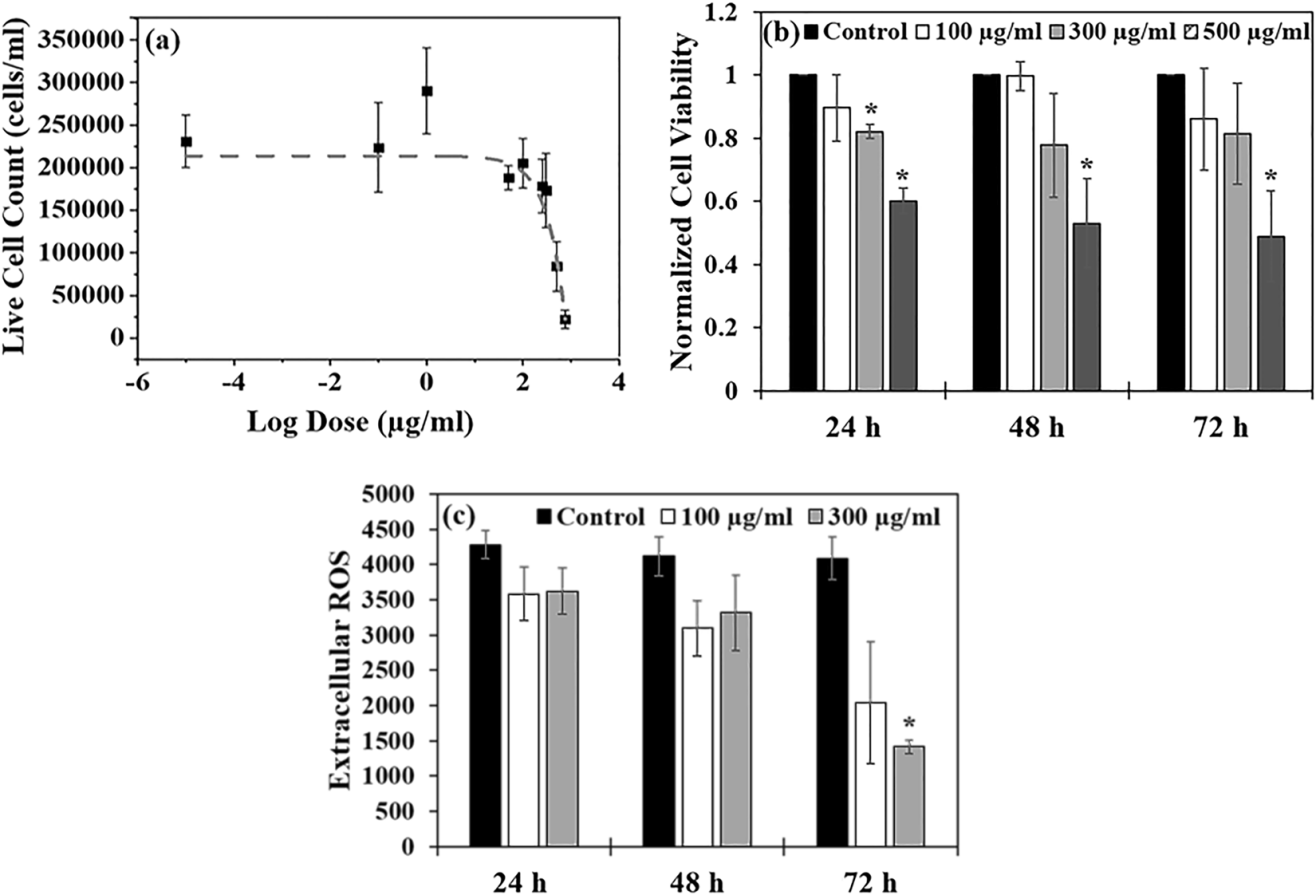
(**a**) Dose response curve (based on live cell counts) for BEAS-2B cells exposed to PLACC900 from 0–750 µg/ml (n = 5). The data was fit via a sigmoidal curve using OriginPro (OriginLab Corporation) software. (**b**) Cellular viability (based on WST assay) for cells exposed to PLACC900 (n = 6). The symbol * indicates a significant difference between the control cells and exposed cells. The values are normalized relative to the controls. (**c**) Extracellular ROS of cells exposed to varying doses of PLACC900 (n = 4). The symbol * indicates a significant difference between the control cells and exposed cells. Significance was determined by one-way analysis of variance ANOVA with p < 0.05, * indicating significance.

A dose response curve was initially performed to identify the PLACC900 concentrations that will create a differential effect on the cell viability. Specifically, cells were exposed to doses of 0.1, 1, 50, 100, 250, 300, 500, and 750 µg/ml for 24 h (Fig. 4a). The large number of doses was chosen to mimic what a worker might inhale in areas of disposal, where it is known that concentrations could vary based on the point of emission, time of day, the amount and incorporation of the nanoparticle in the material being disposed, as well as the amount of the material being disposed, respectively^43,116,117^. Additionally, such doses represent different working lifetimes by taking into account total work hours, and particle and lung characteristics of the worker^118^.

Analysis showed that the resulting IC_50_ value (i.e., concentration of PLACC900 required to inhibit cell growth by 50%) of PLACC900 was 435 µg/ml. No significant decrease in cell viability was observed for cells exposed to 100 µg/ml (below IC_50_) over the 72 h (Fig. 4b). However, after 24 h of exposure a significant decrease in cellular viability (around 20 and 50%) was observed when BEAS-2B cells were exposed to PLACC900 at 300 and 500 µg/ml, respectively. This effect continued for BEAS-2B cells exposed to 500 µg/ml PLACC900 throughout the 72 h of exposure. When examining the effect of doses under the IC_50_ value of PLACC900 over time on cellular proliferation, there were not any significant decreases even after 72 h of exposure (Supplementary Fig. S4).

The decrease in cellular viability could be due to the accumulation of reactive oxygen species (ROS) and the effects that such accumulation could induce on the cells^119^. In particular, previous studies have showed that CC by itself could induce internal ROS^69^ to lead to cellular membrane damage and cell morphology changes from an oval to a more circular profile^120,121^. Our results showed that the cells exposed to 300 µg/ml PLACC900 had a significant decrease in extracellular ROS after 72 h of exposure, indicating that ROS may be building up within individual cells^119^, to potentially cause damage to internal organelles or cell membrane and shape^46^ (Fig. 4c). In our experiments, cells were only exposed to doses below the IC_50_ in order to ensure that the cell population would be high enough to produce observable extracellular ROS (control measurements were from live cells). The observed error bars are attributed to the byproducts interaction with the reagent^122^ or to the variability in the surface morphology of the byproduct^38^. Indeed, when examining the byproduct itself (no cells), both doses showed to produce more extracellular ROS relative to the media alone, though this effect was only significant at 72 h (Supplementary Fig. S5).

**Figure 5:**
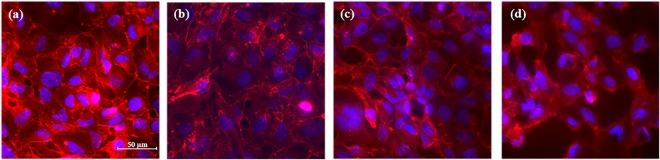
Fluorescent images of the cell membrane (red) and nucleus (blue) for (**a**) control cells and cells exposed to PLACC900 at (**b**) 100 µg/ml, (**c**) 300 µg/ml, and (**d**) 500 µg/ml after 24 h.

Cellular imaging complemented the above results showing a dose-dependent behavior for cells exposed to PLACC900 at 100, 300, and 500 µg/ml over a 24 h period. For these observations, the plasma membrane was stained red and nucleus blue (Fig. 5a–d). Analysis showed that at 24 h of exposure, the control and cells exposed to 100 µg/ml displayed a confluent monolayer with oval cells. However, the cells were no longer confluent upon exposure to doses of 300 µg/ml and higher. Further, the cells seemed to assume irregular shapes relative to the controls, with stretched or circular profiles being noted. Cells exposed to 500 µg/ml PLACC900 displayed the greatest loss in cell monolayer.

**Figure 6:**
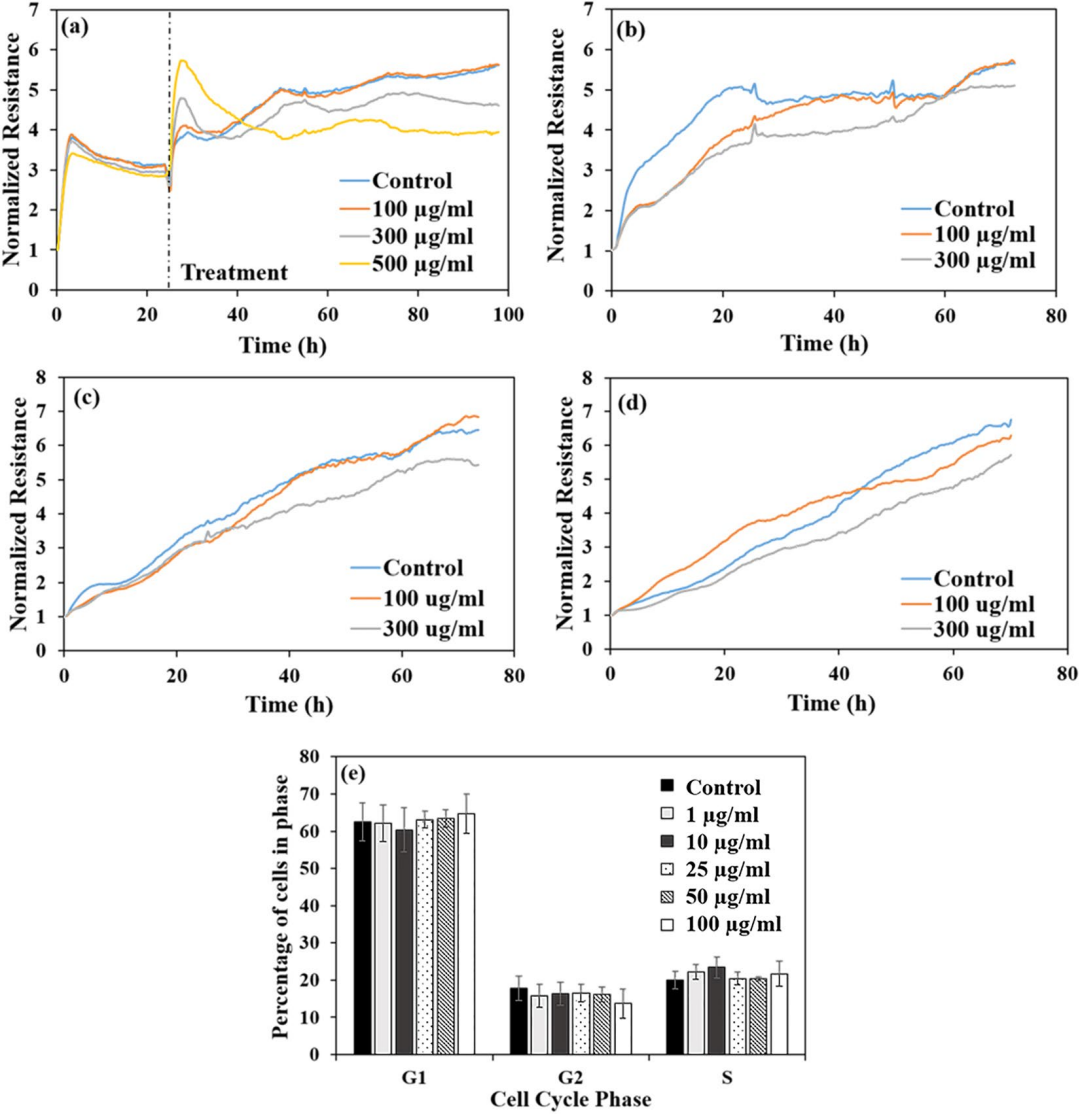
(**a**) Representative real-time measurements of normalized resistance for BEAS-2B cells before and during exposure to PLACC900 from 100–500 µg/ml. Representative real-time measurements of normalized resistance for the recovery of BEAS-2B cells over 72 h after exposure to PLACC900 for (**b**) 24 h, (**c**) 48 h, and (**d**) 72 h. (**e**) Percentage of cells in the G1, G2, or S phase of the cell cycle after exposure to 1–100 µg/ml PLACC900 (n = 4).

The observed change in shape as well as the loss of the cellular monolayer and buildup of ROS could indicate that cells may have begun to lose their ability to attach to substrates, as well as to other cells, two mechanisms hinting at deleterious effects and potential toxicity^120,121,123,124^ of the byproducts. Indeed, our electrical cell-substrate impedance sensing (ECIS) analysis indicated that cells exposed to PLACC900 at 100, 300, and 500 µg/ml and subsequently monitored for 72 h (Fig. 6a) had changes in their resistance pathways which were both time and dose dependent. ECIS is known to monitor changes in cell-cell and cell-substrate interactions, cell morphology, and coverage in real time^125,126^, with such changes being quantitatively analyzed, at a nanoscale resolution, and in a non-invasive^122,127^, and high-throughput manner^125,126,128,129^. Specifically, while cells exposed to 100 µg/ml had very similar resistance values relative to the control over the whole exposure time, cells exposed to 300 and 500 µg/ml of the byproducts displayed an initial increase in resistance, with the increase being more dramatic and longer for the 500 µg/ml dose. However, after 24 h of exposure, the resistances lowered, again, all relative to the control cells. Such drops in resistance complement the observed decreases in cell viability, proliferation, and monolayer coverage. Additionally, changes in cell shape, especially, from a stretched, spread cell to a more rounded one with less contact with the electrode surface could explain the decreased resistances^125,126^.

The recovery of cells exposed to PLACC900 was also non-invasively monitored in order to determine if any of the observed effects lasted. Only 100 and 300 µg/ml doses were again used in order to allow for an adequate number of cells to be added to the electrodes. Overall, the cells ability to recover was dose dependent. Specifically, analysis showed that after 24 h of exposure to 100 and 300 µg/ml of PLACC900, the cells showed lower resistance values relative to the control over their first 24 h of recovery (Fig. 6b). However, cells exposed to 100 µg/ml PLACC900 had similar resistances to the control within 48 h of recovery, while cells exposed to 300 µg/ml had similar resistances to the control within 60 h of recovery. After a 48 h exposure to PLACC900, cells exposed to 100 µg/ml also had slightly lower resistance values relative to the control again over the first 48 h of recovery, but eventually had similar resistances to the control cells by 40 h of recovery (Fig. 6c). Cells exposed to 300 µg/ml of PLACC900 had slightly lower resistances relative to the control cells over the full 72 h of recovery. Finally, after 72 h of exposure to the byproduct, cells exposed to 100 µg/ml had higher resistances relative to the control for the first 40 h of recovery, while cells exposed to 300 µg/ml had lower resistances relative to the control over the full 72 h of recovery (Fig. 6d). Similar to our results, AshaRani *et al*. noted that cells exposed to 400 µg/ml concentrations of silver nanoparticles took a month to recover when compared to cells exposed to 100 or 200 µg/ml silver nanoparticles which were able to recover completely within 5 or 15 days, respectively^130^.

The ability of the cells to recover was also confirmed by cell cycle analysis (Fig. 6e) with analysis showing that there were no significant differences in cell cycle phases for G1, G2, or S after exposure to any of the doses, all relative to the control cells. While previous studies have shown genotoxic effects of nanoclays, ranging from DNA strand breaks^67^ to condensed chromatin^131^ and micronuclei^132^, as well as, changes in gene expression^132^, the lack of cell cycle arrest and normal progression through the cell cycle for cells exposed to PLACC900 hints at DNA stability^133^ and lack of DNA damage^134^ at lower doses.

The observed recovery after removal of the exposure could be attributed to a volume-based dilution of the internalized byproducts of PLACC900 and/or lower toxicity that such byproducts have on the cell. For the first, previous analysis showed that internalized gold nanoparticles for instance were devised between the surviving cells to lead to a cell recovery profile dependent on the cell growth and division^135^. However, in such studies, the nanoparticles were around 45 and 13 nm, much smaller relative to the sizes recorded for PLACC900^135^. For the second, PLACC900 seems to be following a similar toxicity profile to thermally degraded nanoclays which have previously been shown to be less toxic relative to their as-received counterparts^40,41^. Previous results focused on nanoclays alone also showed wide ranges in toxicity with IC_50_ values as low as around 1 µg/ml^39^ to more than 1000 µg/ml^136^, generally with the organic modifier being the cause of the toxicity^39,67–69^. In particular, thermally degraded byproducts of one pristine and three organically modified Nanomer nanoclays had higher IC_50_ values (indicating lower toxicity) relative to their as-received counterparts when exposed to two types of lung cells^41^. The lower toxicity effects were attributed to loss of the organic modifier, along with changes in morphology, size, and molecular and elemental composition of the byproduct when compared to the as-received one. Some changes may however exist herein for the nanocomposite when compared to the nanoclay most likely due to morphology, size, or potential polymer species trapped within the nanocomposite byproduct. For instance, while some of the smaller PLACC900 particles may be internalized^137,138^, it is likely that PLACC900 causes deleterious effects at the cell membrane level, disrupting the present macromolecules and potentially causing membrane damage^87^ or causing rupturing due to its uneven, jagged profile as displayed by SEM^86,87^, however with such damage to be recovering in a time-dependent manner as shown by our real-time analysis. Additionally, the variability observed in toxic effects may be due to the variable surface morphology and size of the byproducts. Finally, PLACC900 itself may have effects similar to that of crystalline silica, i.e., inflammation and collagen deposition, since it contains similar CC900 properties previously shown to produce a low, persistent inflammation profile in mice^52^.

## Conclusions

PLACC900 byproducts were obtained by incineration of a PLA-based nanocomposite reinforced with methyl, tallow, bis-2-hydroxyethyl, quaternary ammonium montmorillonite (CC). Characterization of PLACC900 showed a loss of CC’s organic modifier and the majority of the polymer matrix, as well as the appearance of two different surface morphologies attributed to the uneven dispersion of CC enforcing the PLA. Toxicity in an *in vitro* human lung epithelial cell line only occurred at higher end doses with the majority of the cells displaying the ability to recover when exposed at low doses. Specifically, the toxic effects of PLACC900 generally did not appear until the dose of 300 µg/ml, with doses at and above 300 µg/ml causing decreases in cellular viability and coverage, as well as alterations to cellular morphology and to the cytoskeleton. While more information is required to determine mechanisms for nanocomposites degradation and ultimately toxicity of their end of life cycle byproducts, proper engineering control and protocols for workers in areas of nanocomposite disposal should be implemented to help lessen their inhalation exposure to high doses of thermally degraded byproducts.

## Methods

### Nanocomposite and Incinerated Byproducts Preparation

Cloisite 30B (CC) was obtained from Southern Clay Products (Gonzales, TX) and, per the manufacturer specifications, organically modified via an ion-exchange reaction with methyl, tallow, bis-2-hydroxyethyl, quaternary ammonium (Scheme S1) at a concentration of 90 meq/100 g clay. Polylactic acid 6752 (PLA; NatureWorks) was melt-mixed with CC loaded at a 5 wt. %, in a Thermo-Haake internal mixer operating at 200 °C and 80 rpm for 5 min. Thin films were then molded at 200 °C using a compression press to form PLA-CC nanocomposites (PLACC), as well as PLA films to be used as controls.

Samples of PLA and PLACC (1 g per sample) were thermally degraded using a TGA701 Thermogravimetric Analyzer (LECO) to mimic their disposal. To determine the moisture content, the samples were heated in nitrogen at a rate of 6 °C/min and in a range of temperatures from 25 °C to 105 °C. To determine the volatile content, the samples were heated from 105 °C to 950 °C, also in nitrogen and at a rate of 43 °C/min. Finally, to determine the ash content, the samples were heated from 550 °C to 900 °C in oxygen, at a rate of 15 °C/min. The resulting ash was collected to serve as a model of the byproducts resulted from incineration i.e., thermally degraded PLA-CC nanocomposite (PLACC900).

### Materials Characterization

Elemental composition and surface morphology of PLA, PLACC, and PLACC900 were investigated using a Hitachi S-4700 Field Emission Scanning Electron Microscope (SEM, Hitachi High-Technologies Corporation) equipped with energy dispersive X-ray (EDX) spectroscopy. Surface morphology was examined at 5.0 kV while elemental composition was determined at 20.0 kV. For the analyses, dry films or powder samples (ca. 10–15 mg) were mounted onto a carbon tape and then sputter coated with gold/palladium for 10 s in vacuum injected with argon. The argon atoms were ionized and collided with the gold/palladium target, causing the metal ions to deposit on the sample in a thin conductive layer of about 3 nm, as calculated using the equation

$${\rm{d}}={\rm{kIVt}},$$

where d is thickness, k is a constant value of 0.17, I is plasma current, V is voltage, and t is the time. For the EDX analysis, data was obtained from 5 different areas of each respective sample portion used.

Molecular composition of the samples (PLA, PLACC, and PLACC900) was determined using Fourier Transform Infrared Spectroscopy (FTIR, Digilab FTS 7000) equipped with diamond Attenuated Total Reflection (ATR). Scans were collected in the range of 4000–400 cm^−1^ at a resolution of 4 cm^−1^; a total of 100 scans were co-added to form the final spectrum for each of the samples.

The crystallinity of PLA and PLACC and the degree of exfoliation of CC in PLACC was determined via X-ray diffraction (XRD). Specifically, PANalytical X’Pert Pro XRD (PANalytical) was used to determine crystallinity via a Cu-kα1 8047.2 eV source at 45 kV and 40 mA with a 10 sec/step in a 5–80° 2θ range. Bruker D8 Discovery XRD (Bruker) was used to determine the degree of exfoliation of CC in PLACC; thin films were mounted on the sample holder and diffraction was obtained in the 2θ range of 1–10° at an increment of 0.02° and scan speed of 10 sec/step via a Cu-kα1 8047.2 eV source at 40 kV and 40 mA. Basal spacing was determined by Bragg’s equation

$$n{\rm{\lambda }}=2\mathrm{dsin}\,{\rm{\theta }},$$

where n is an integer, λ is the wavelength of the X-ray radiation (0.1546 nm), d is the spacing between lattice planes, and θ is the measured diffraction angle.

The absorption spectra for PLA and PLACC was determined in the range of 200–800 nm via the Shimadzu UV-Vis spectrophotometer (Shimadzu Scientific Instruments). UV barrier properties of the film were determined by measuring transmission at 280 nm, and transparency of the films was determined by measuring transmission at 660 nm, also via the Shimadzu UV-Vis spectrophotometer.

The tensile strength, Young’s Modulus, and elongation at break for films of PLA and PLACC were evaluated via the Instron E1000 (Instron Corporation) under a 2 kN load cell and using the Bluehill 3 software. For this, rectangular films of PLA and PLACC, 5 mm in width × 32 mm in length × 0.3 mm in thickness, were placed in the Instron grips, and the experiments were performed with a crosshead speed set at 5 mm/min. A specimen gauge length of about 25 mm was used for each sample upon gripping in the crosshead.

The size distribution of PLACC900 was determined by dynamic light scattering (DLS) via the Mastersizer 2000 with a Hydro 2000S accessory (Malvern Instruments). For this, solutions of PLACC900 dispersed and bath sonicated in cell culture media (Dulbecco’s Modified Eagle Medium: DMEM) containing 5% fetal bovine serum (FBS), 1% L-glutamine, and 1% penicillin-streptomycin or in phosphate buffered saline (PBS) were dropped into the Hydro 2000S until laser obscuration was within 10–20%. The size analysis was performed 3 consecutive times with a stirrer speed of 1750 rpm and under continuous sonication. The media and sonication conditions were chosen to mimic the cell exposure studies.

### Cell Culture

Immortalized human bronchial epithelial (BEAS-2B) cells were cultured in DMEM media containing 5% FBS, 1% L-glutamine, and 1% penicillin-streptomycin (all reagents were purchased from Life Technologies). The cells were passaged regularly using 0.25% trypsin (Invitrogen) and incubated at 37 °C, 5% CO_2_, and 80% relative humidity. Before each experiment cells were grown to a confluent monolayer.

### Dose Response Curve (IC_50_)/ Cell Viability

BEAS-2B cells were seeded in a 12 well plate (Falcon) at a density of 2.0 × 10^5^ cells/ml. After 24 h, the cells were exposed to PLACC900 from 0–750 µg/ml, with the doses obtained by serial dilutions. For this, samples were first sonicated for 8–10 min in media by using a bath sonicator (Branson). After 24 h of exposure to PLACC900, the cells were trypsinized and stained with 0.4% trypan blue solution (Invitrogen). Subsequently, 10 µl of the sample containing the stained cells was added to a hemocytometer (Hausser Scientific), and the number of cells in the 4 outer grids was counted through use of the Leica DM IL optical microscope (Leica Microsystems) using a 10X objective. Cell counting was performed via the hemocytometer and microscopy to allow for proper distinction between cells and any remaining byproduct present in the cellular suspension. OriginPro (OriginLab Corporation) software was used to determine the IC_50_ value via fit with a sigmoidal curve.

In another assay, BEAS-2B cells were seeded in a 96 well plate (CellTreat Scientific Products) at a density of 2.0 × 10^5^ cells/ml. After 24 h, the cells were exposed to PLACC900 at 100, 300, and 500 µg/ml dispersed in media following 8–10 min sonication. Cells in only media served as controls. The 4-[3-(4-Idophenyl)-2-(4-nitrophenyl)-2H-5-tetrazolio]-1,3-benzene disulfonate known as WST-1 assay (Roche, USA) was used to determine cellular metabolic activity since it is known that changes in color of such reagent are produced when cellular dehydrogenases of metabolically active cells reduce it to formazan^139^. Twenty four, 48, and 72 h post exposure to PLACC900, 10 µl of WST was added to the wells. Cells (exposed and control) were incubated for 2 h and absorbance was read at 485 nm using a FLUOstar OPTIMA plate reader (BMG LABTECH). Media and PLACC900 byproduct dispersed in media served as blanks with their absorbance values being subtracted from the cellular measurements counterparts.

### Extracellular Reactive Oxygen Species (ROS)

BEAS-2B cells were seeded in a 12 well plate at a density of 1.5 × 10^5^ cells/ml. After 24 h, the cells were exposed to 100 or 300 µg/ml of PLACC900 dispersed in media as previously described. After 24, 48, and 72 h of exposure, 50 µl of media from each treatment was transferred to a black-bottomed 96 well plate (Corning, Inc.). Subsequently, 50 µl of PBS and 50 µl of Lumigen ECL Plus (Lumigen, Inc.) were added to each well, and the samples were incubated for 5 min in the dark. Luminescence was read at 600 nm via the FLUOstar OPTIMA plate reader. Media as well as PLACC900 dispersed in media, at each dose, served as blanks. Extracellular reactive oxygen species (ROS) was calculated by subtracting PLACC900 luminescence (determined via subtraction of media from the PLACC900 + media blanks) from the respective cellular measurements.

### Cellular Imaging

BEAS-2B cells were seeded on glass coverslips (15 mm diameter; Fisher Scientific) in a 12 well plate at a density of 1.5 × 10^5^ cells/ml overnight. The cells were subsequently exposed to 100, 300, or 500 µg/ml PLACC900 dispersed in media as previously described. After 24 h, the media was removed and the cells were washed two times with Hank’s Balanced Salt Solution (HBSS) (Corning, Inc.), fixed with 4% formaldehyde (Sigma-Aldrich) for 15 min and at 37 °C, and subsequently washed 3 more times with HBSS to remove any remaining formaldehyde. The cells’ plasma membranes and nuclei were then stained with 3 µg/ml Alexa Fluor 594 wheat germ agglutinin (WGA) and 2 µM Hoechst 33342 (Image-iT LIVE Plasma Membrane and Nuclear Labeling Kit, Life Technologies), respectively, both dispersed in HBSS, for 10 min and at 4 °C. After incubation, cells were washed 2 times with HBSS, the cover slides were mounted on glass coverslips, and imaged under a Nikon Inverted Microscope Eclipse Ti Series (Nikon) and a 40X objective. The NIS-Elements BR 3.1 software was used to analyze the size and morphology of the cells.

### Electrical Cell-substrate Impedance Testing

Real-time measurements of BEAS-2B cellular resistance during and after exposure with PLACC900 were performed using an electrical cell-substrate impedance sensing instrument (ECIS-ZΘ, Applied Biophysics, NY). For such cellular studies, a 96 well plate (96W10idf) that contained inter-digitated finger connection electrodes covering an area of about 4 mm^2^ of each well were used. Before addition of the cells, the electrodes were stabilized for 2 h with 200 µl of media to minimize any drift during the experiment.

For exposure, BEAS-2B cells were seeded on the ECIS electrodes at a density of 2.0 × 10^5^ cells/ml in a volume of 150 µl/well. After 24 h, the cells were exposed to 100, 300, or 500 µg/ml of PLACC900, dispersed in media; cells in media served as controls. The resistance of the cells was monitored continuously for 72 h. The recovery of the cells was also monitored for 72 h. For this, parallel experiments were performed in which, after 24, 48, and 72 h of exposure, the cells were trypsinized and counted so that 1.0 × 10^5^ cells/ml could be added to its respective ECIS well at a volume of 150 µl/well.

### Cell Cycle

BEAS-2B cells were seeded in a 6 well plate (Corning, Inc.) at a density of 2.5 × 10^5^ cells/ml; cells in media served as controls. After 24 h, the cells were exposed to 1–100 µg/ml (1, 10, 25, 50, and 100 µg/ml) of PLACC900 dispersed in media as previously described. After 24 h, the cells were washed 2 times with PBS, trypsinized, pelleted, and washed again. The cells were then resuspended and fixed with 70% ethanol overnight at −20 °C. Subsequently, the cells were pelleted and the ethanol decanted. The cells were once again washed and resuspended in 0.2% Tween 20 (Fisher Scientific) for 15 min at 37 °C. In another step, PBS was added and the cells were pelleted and resuspended in 180 µg/ml Ribonuclease A-PBS (Sigma-Aldrich) for 15 min at room temperature. Finally, the DNA of the cells was stained via a 15 min incubation with 75 µg/ml propidium iodide solution (Sigma-Aldrich) at room temperature. After incubation, the volume was brought up with 300 µl of PBS. The cells’ DNA content was then analyzed via the BD LSRFortessa (BD Biosciences) and BDFACSDiva 8.0 software and knowing that the amount of DNA will double in the G2 phase when compared with S phase of the cell cycle. There were 20,000 events contained in the gated area of the live cell population per sample (formed via forward scatter and side scatter) used for analysis.

### Statistical Analyses

All cellular experiments were repeated at least 4 times for all samples, with the exception of cellular imaging which was repeated 3 times. All tables are presented as the average value with (+/−) SD values. All graphs are presented as the mean value of the number of indicated replicates with (+/−) SE bars. Significance was determined by one- or two-way analysis of variance ANOVA with p < 0.05* indicating significance. OriginPro software was used for determination of the IC_50_ value for PLACC900 by using a sigmoidal dose response fit on the average of the 5 replicates.

### Disclaimer

The findings and conclusions in this report are those of the author(s) and do not necessarily represent the official position of the National Institute for Occupational Safety and Health, Centers for Disease Control and Prevention.

## Electronic supplementary material

Supplementary Information
