# Supplementary material for: Incineration of Nanoclay Composites Leads to Byproducts with Reduced Cellular Reactivity
Source: Sci Rep. 2018 Jul 16;8:10709. doi: 10.1038/s41598-018-28884-y (PMC6048035; doi:10.1038/s41598-018-28884-y)
Supplement: Supplementary file 1 — Supplementary Information [file 41598_2018_28884_MOESM1_ESM.pdf]

Supplementary Information

Incineration of Nanoclay Composites Leads to Byproducts with Reduced Cellular Reactivity

Alixandra Wagner[a], Andrew P. White[a], Man Chio Tang[a], Sushant Agarwal[a], Todd A. Stueckle[b], Yon Rojanasakul[c], Rakesh K. Gupta[a], and Cerasela Zoica Dinu[a*]

[a] Department of Chemical and Biomedical Engineering, West Virginia University, Morgantown, WV 26506, USA
[b] Health Effects Laboratory Division, National Institute for Occupational Safety and Health, Morgantown, WV 26505, USA
[c] Department of Basic Pharmaceutical Sciences, West Virginia University, Morgantown WV, 26506, USA

***Corresponding author:**

Cerasela Zoica Dinu, Ph.D.
Department of Chemical and Biomedical Engineering
West Virginia University
Benjamin M. Statler College of Engineering and Mineral Resources
PO Box 6102
Morgantown, WV, 26506, USA
E-mail: cerasela-zoica.dinu@mail.wvu.edu
Tel.: +1 304 293 9338
Fax: +1 304 293 4139

Methods

Nanocomposite Preparation

Polylactic acid 6752 (PLA) was melt-mixed with Cloisite 30B (CC) loaded at a 5 wt. %, in a Thermo-Haake internal mixer operating at 200 ºC and 80 rpm for 5 min. Thin films were then molded at 200 ºC using a compression press to form PLA-CC nanocomposites (PLACC), as well as PLA films to be used as controls.

Thermal Degradation of PLACC and CC

PLACC (1 g per sample) and CC (0.5 g per sample) were thermally degraded using a TGA701 Thermogravimetric Analyzer (LECO). To determine the moisture content, the samples were heated in nitrogen at a rate of 6 ºC/min and in a range of temperatures from 25 ºC to 105 ºC. To determine the volatile content, the samples were heated from 105 ºC to 950 ºC also in nitrogen and at a rate of 43 ºC/min. Finally, to determine the ash content, the samples were heated from 550 ºC to 900 ºC in oxygen, at a rate of 15 ºC/min.

Material Characterization of PLA, PLACC, and Associated Nanoclays or Byproducts

Molecular composition of CC, thermally degraded CC (CC900), and thermally degraded PLACC (PLACC900) was determined using Fourier Transform Infrared Spectroscopy (FTIR, Digilab FTS 7000) equipped with diamond Attenuated Total Reflection (ATR). Scans were collected in the range of 4000-400 cm^{-1} at a resolution of 4 cm^{-1}; a total of 100 scans were co-added to form the final spectrum for each of the samples. Elemental composition of PLACC900 and CC900 was investigated using a Hitachi S-4700 Field Emission Scanning Electron Microscope (SEM, Hitachi High-Technologies Corporation) equipped with energy dispersive X-ray (EDX) spectroscopy at 20.0 kV.

The absorption spectra for PLA and PLACC was determined in the range of 200-800 nm via the Shimadzu UV-Vis spectrophotometer (Shimadzu Scientific Instruments). UV barrier properties of the film were determined by measuring transmission at 280 nm, and transparency of the films was determined by measuring transmission at 660 nm, also via the Shimadzu UV-Vis spectrophotometer. The tensile strength, Young's Modulus, and elongation at break for films of PLA and PLACC were evaluated via the Instron E1000 (Instron Corporation) under a 2 kN load cell and using the Bluehill 3 software. For this, rectangular films of PLA and PLACC, 5 mm in width x 32 mm in length x 0.3 mm in thickness, were placed in the Instron grips, and the experiments were performed with a crosshead speed set at 5 mm/min. A specimen gauge length of about 25 mm was used for each sample upon gripping in the crosshead.

The size distribution of PLACC900 was determined by dynamic light scattering (DLS) via the Mastersizer 2000 with a Hydro 2000S accessory (Malvern Instruments). For this, solutions of PLACC900 dispersed and bath sonicated in cell culture media (Dulbecco's Modified Eagle Medium: DMEM) containing 5% fetal bovine serum (FBS) or in phosphate buffered saline (PBS), were dropped into the Hydro 2000S until laser obscuration was within 10-20 %. The size analysis was performed 3 consecutive times with a stirrer speed of 1750 rpm and under continuous sonication. To note: DLS predicts size measurements of particles by applying models to the light that is being captured from the scattering of the particles. Specifically, the Mie theory is used to interpret the scattering patterns of light by the particles and assumes that particles are perfect spheres. In order to account for any changes in shape, the Mastersizer 2000 uses the technique known as "equivalent spheres" and instead of measuring the size of the particles it measures the volume they occupy.[3]

Cell Culture

Immortalized human bronchial epithelial (BEAS-2B) cells were cultured in Dulbecco's Modified Eagle Medium (DMEM) containing 5% FBS, 1 % L-glutamine, and 1 % penicillin-streptomycin (all reagents were purchased from Life Technologies). The cells were passaged regularly using 0.25 % trypsin (Invitrogen) and incubated at 37 ºC, 5 % CO_2, and 80 % relative humidity. Before each experiment cells were grown to a confluent monolayer.

Live Cell Count

BEAS-2B cells were seeded in a 12 well plate (Falcon) at a density of $2.0x10^5$ cells/ml. After 24 h, the cells were exposed to PLACC900 at 100 and 300 µg/ml, which were first sonicated for 8-10 min in media via a bath sonicator (Branson). Cells in only media served as controls. Twenty four, 48, and 72 h post exposure to PLACC900 the cells were trypsinized and stained with 0.4% trypan blue solution. Subsequently, 10 µl of the sample containing the stained cells was added to a hemocytometer, and the number of cells in the 4 outer grids were counted through use of the Leica DM IL optical microscope using a 10X objective.

Reactive Oxygen Species (ROS) Generation of PLACC900

PLACC900 was dispersed in media via a bath sonicator at doses of 100 and 300 µg/ml. The solutions were then placed in a 12 well plate with media only serving as the control. After 24, 48, and 72 h of incubation at 37 ºC, 5 % CO_2, and 80 % relative humidity, 50 µl of the PLACC900+media from each dose (and media only as control) was transferred to a black-bottomed 96 well plate. Subsequently, 50 µl of PBS and 50 µl of Lumigen ECL Plus (Lumigen, Inc.) were added to each well, and the samples were incubated for 5 min in the dark. Luminescence was read at 600 nm via the FLUOstar OPTIMA plate reader.

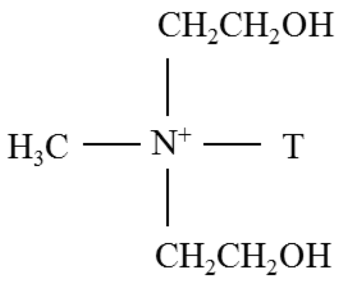

Supplementary Scheme 1: Organic modifier of CC; methyl, tallow, bis-2-hydroxyethyl, quaternary ammonium.

Results

Tables:

Supplementary Table S1: UV dispersion (determined by Transmittance at 280 nm (T_{280nm})) and Transparency (determined by Transmittance at 660 nm (T_{660nm})) of PLA and PLACC. The symbol * indicates a significant difference between PLA and PLACC (n=5).

	T_{280nm}	T_{660nm}
**PLA**	3.85 +/- 0.12	4.88 +/- 0.08
**PLACC**	3.35 +/- 0.21*	7.11 +/- 0.20*

Supplementary Table S2: Mechanical properties of PLA and PLACC as determined via the Instron. The symbol * indicates a significant difference between PLA and PLACC (n=5).

	Tensile Strength (MPa)	Elongation at Break (mm)	Young's Modulus (MPa)
**PLA**	81.2 +/- 19.0	0.436 +/- 0.093	9291.3 +/- 850.9
**PLACC**	66.8 +/- 22.3	0.270 +/- 0.053*	11055.8 +/- 1517.6*

Material Characterization

Molecular composition of CC, CC900, and PLACC900 was determined by Fourier Transform Infrared Spectroscopy (FTIR) (Supplementary Fig. S1a). The nanoclay, CC, displayed peaks at 1000 cm^{-1}, indicative of Si-O-Si stretching deformation,[1] and at 900 and 840 cm^{-1}, indicative of Al-OH-Al deformation of aluminates[2] and deformation of OH linked to Al^{3-} and Mg^{2-},[2] respectively. The peaks associated with the organic modifier (2920, 2850, and 720 cm^{-1})[1,2] were no longer present in the spectra of CC900 and PLACC900. CC900 and PLACC900 displayed a similar spectra, indicating similar molecular compositions with the only major peak occurring around 1000 cm^{-1}. Elemental composition was determined by energy dispersive X-ray (EDX) spectroscopy (Supplementary Fig. S1b). There were no significant differences between PLACC900 and CC900, which both showed elements commonly associated with montmorillonite (MMT) nanoclay, such as silicon, oxygen, magnesium, and aluminum.

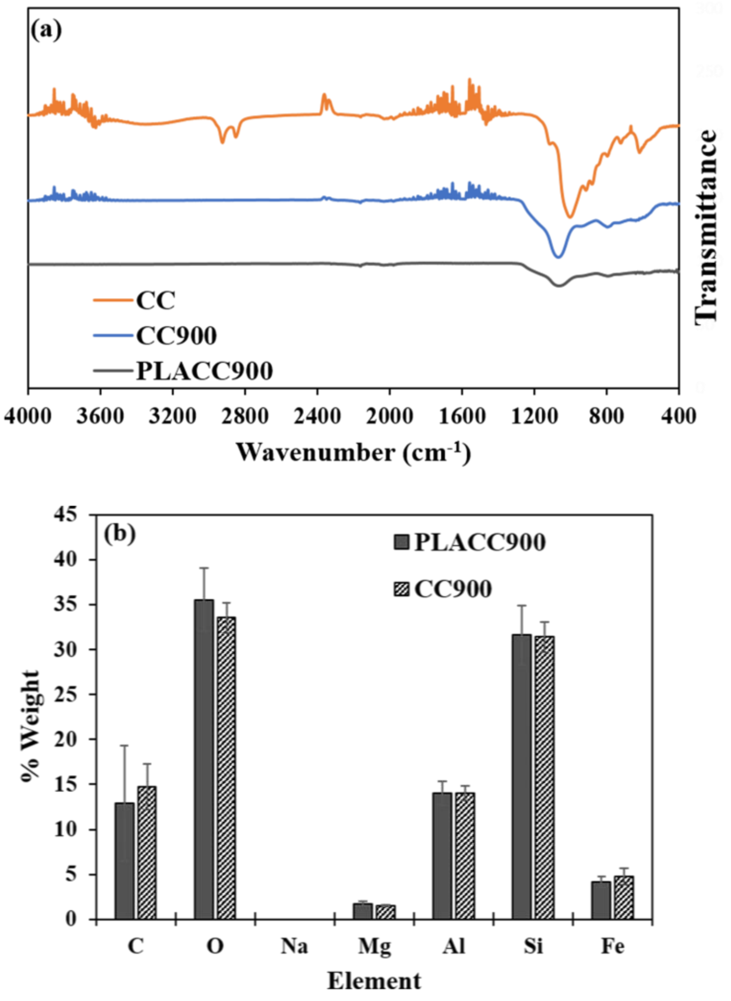

Supplementary Figure S1: (a) FTIR spectra for CC, CC900, and PLACC900 (n=2). (b) Elemental composition of PLACC900 and CC900 as determined by EDX (n=5).

The films of PLA and PLACC displayed similar absorbance spectra relative to each other with peaks around 245 and 270 nm, respectively (Supplementary Fig. S2a). The nanocomposite, PLACC, displayed a different color relative to the control film of PLA, likely due to the incorporation of the CC (Supplementary Fig. S2b,c).

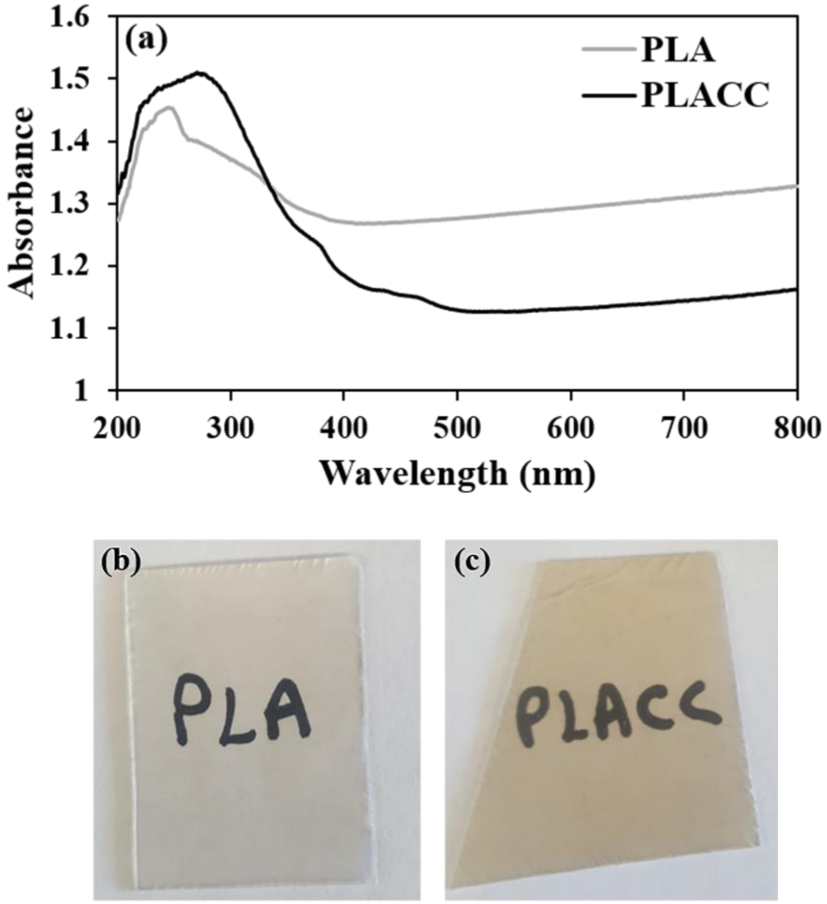

Supplementary Figure S2: (a) Absorbance spectra for PLA and PLACC from 200-800 nm (n=3). Representative films of (b) PLA and (c) PLACC.

In order to measure the size distribution of PLACC900, the particles were sonicated and size measurements obtained via the Matersizer 2000 DLS system (Supplementary Fig. S3). The byproduct, PLACC900, displayed a size distribution in the micrometer range with similar distributions when dispersed either in cellular media or the control buffer, PBS.

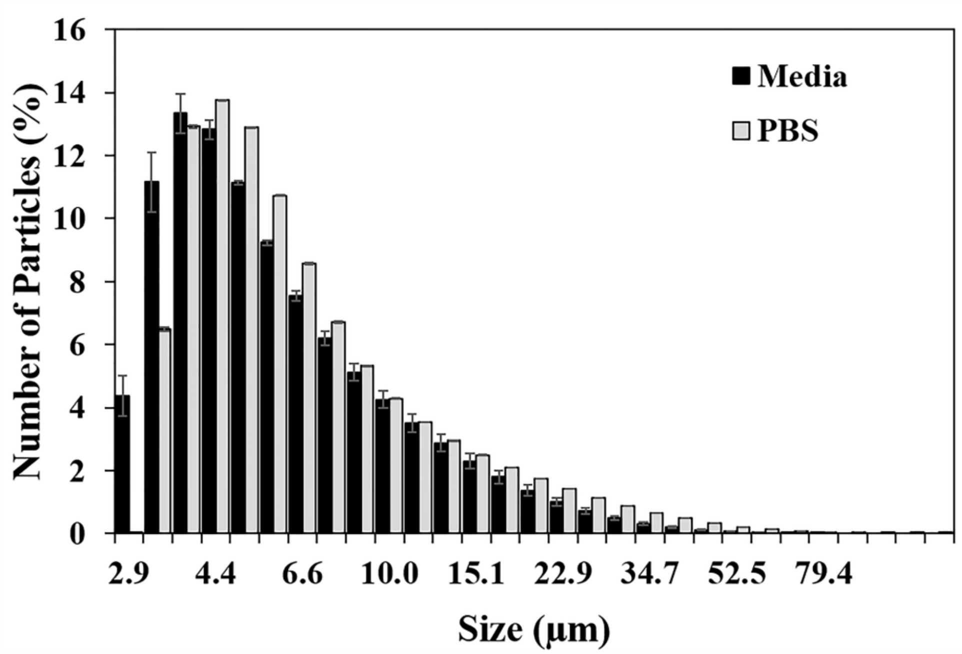

Supplementary Figure S3: Average particle diameter size distribution of PLACC900 in cellular media or control buffer, PBS (n=3).

2.2 Toxicity Analyses

BEAS-2B cells were exposed to 100 and 300 µg/ml of PLACC900 over 72 h. No significant differences in live cell count were obtained between the control cells (cells in only media) and the exposed cells over the 72 h of exposure.

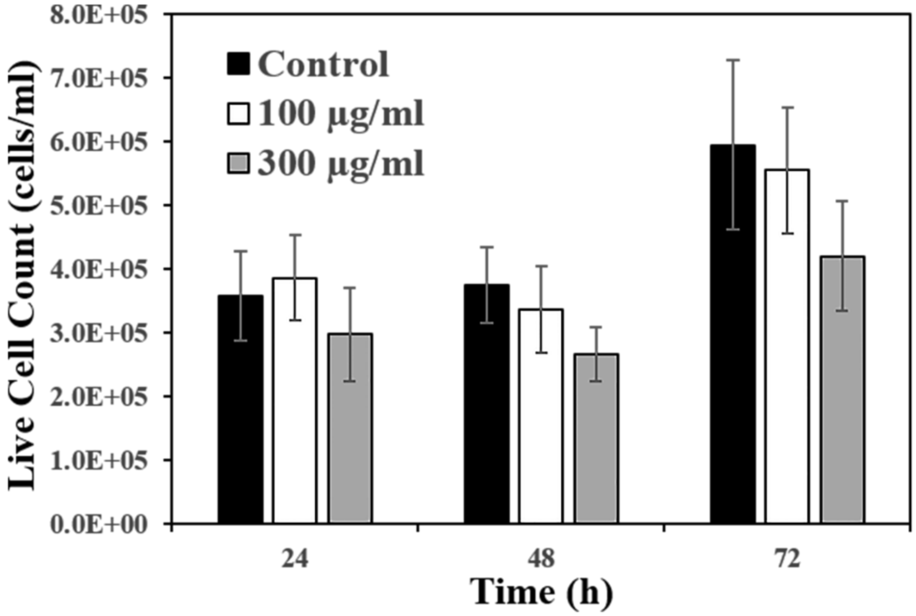

Supplementary Figure S4: Live cell count of BEAS-2B cells exposed to PLACC900 over 72 h (n=6).

In order to determine if the particles of PLACC900 were producing reactive oxygen species (ROS) their luminescence was measured when dispersed in media over 72 h. No significant differences were obtained until 72 h in which both 100 and 300 µg/ml PLACC900 produced a higher luminescence relative to the control (only media), indicating an excess production of ROS.

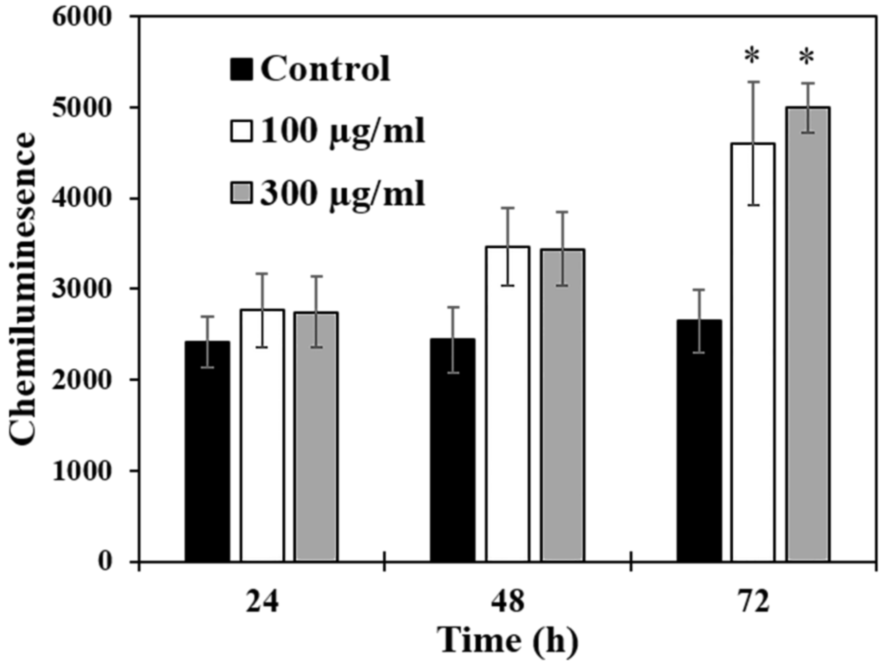

Supplementary Figure S5: ROS generation of PLACC900 at varying doses in DMEM (n=6). The symbol * indicates a significant difference between the control and PLACC900.

References

1. Lordan, S., Kennedy, J.E. & Higginbotham, C.L. Cytotoxic effects induced by unmodified and organically modified nanoclays in human hepatic HepG2 cell line. *J. Appl. Toxicol.* **31**, 27-35 (2011).

2. Saikia, B.J. & Parthasarathy, G. Fourier Transform Infrared Spectroscopic characterization of kaolinite from Assam and Meghalay, Northeastern India. *J. Mod. Phys.* **1**, 206-210 (2010).

3. Malvern Instruments. Masersizer 2000 User Manual. MAN0384, Issue 1.0 (2007).
